# Supplementary material for: Applying qualitative methods to experimental designs: A tutorial for the behavioral sciences
Source: PLoS One. 2025 Jun 16;20(6):e0324936. doi: 10.1371/journal.pone.0324936 (PMC12169552; doi:10.1371/journal.pone.0324936)
Supplement: Appendix A — The translated version of the observation list as was used during the control weeks (PDF) [file pone.0324936.s001.pdf]

Appendix A: Observation list control week. A Dutch version was used during data collection, with a table per half hour. We offer the translated example for readability.

We filled in the observation list to show an example of what kind of data was observed. In this example, the researcher observed from 10 to 10.30h, and saw a total of 11-20 people in that timeframe. They conducted four observations, e.g. Person 1 who actively avoided some people, but did not continuously keep 1.5m distance, or Person 3 wears a face mask and does not encounter others (therefor neither does nor doesn't keep distance). Field notes, rightmost column, give more details on the observed people.

Table 1: ***Example of filled in observation list, not actual data*:**

Name observant
Location observation:
☐ square outside a college hall ☐ main entrance of a lecture hall ☒ entrance to lecture rooms

Time period	Number of people at the location	Person observed	Wears face mask	Keeps 1.5m distance	Addresses others to keep their distance	Avoids people actively to keep their distance	Uses hand sanitizer	Does not keep distance	Does not keep distance to friends	Does not keep distance to strangers	Field notes
10:00-10:30	☐ 0-10	1	1	1		1					1. F, walks in and runs into two people who are standing around talking. Waits for that before walking on.<br>2. F, walks in with girlfriend. Have both put on mouthpiece before entering. Uses alcohol dispenser and keeps distance when girlfriend uses it. Then walks in walking direction upstairs. Otherwise few people in the room.<br>3. M, is alone. Wears mouth mask and uses alcohol dispenser. Walks through to the cafeteria.<br>4. M, is with a colleague. Does not wear mouth mask over nose. Colleague does wear mouth mask properly. Does not keep distance from each other while walking. Moves a little further apart when they see the security guard.
	☒ 11-20	2	2				2			2	
	☐ 21-30	3	3								
	☐ 31-40	4						4	4		
	☐ 41-50										
	☐ 50+										
