# Supplementary material for: Applying qualitative methods to experimental designs: A tutorial for the behavioral sciences
Source: PLoS One. 2025 Jun 16;20(6):e0324936. doi: 10.1371/journal.pone.0324936 (PMC12169552; doi:10.1371/journal.pone.0324936)
Supplement: Appendix B — The translated version of the observation list as was used during the intervention weeks. (PDF) [file pone.0324936.s002.pdf]

Appendix B: Observation list intervention weeks. A Dutch version was used during data collection, with a table per half hour. We offer the translated example for readability.

We filled in the observation list to show an example of what kind of data was observed. In this example, the researcher observed from 8.30 to 9h, and saw a total of 21-30 people in that timeframe. They conducted three observations, e.g. Person 1 who actively avoided some people, but did not continuously keep 1.5m distance, or Person 2 who did not keep their distance at all. Field notes, rightmost column, give more details on the observed people.

Table 1: ***Example of filled in observation list, not actual data*:**

Name observant
Location observation:
☒ square outside a college hall ☐ main entrance of a lecture hall ☐ entrance to lecture rooms

Time period	Number of people at the location	Person observed	Wears face mask	Keeps 1.5m distance	Addresses others to keep their distance	Avoids people actively to keep their distance	Uses hand sanitizer	Does not keep distance	Does not keep distance to friends	Does not keep distance to strangers	Notices interventions	Stands still in front of intervention	Looks at intervention	Discusses intervention with others	Field notes
10:00-10:30	☐ 0-10	1				1		1		1					1. F, was alone, crosses the square. there are 2 long rows at the food stalls, at one she walks around it with a short turn. The second row extends to the library so she cannot go around it and has to go through it. She quickens her stride as she crosses through the row and bows her head down, the back man in the row takes a small step back to give her space but still they do not keep 1.5m distance.<br>2. F, arrives with friend, they cross the square, go through 1 of the rows in front of the food stalls to get to the second one and join the back. She walks and stands close to her friend while they are busy talking. She does not keep 1.5m distance from the person in front of her in the row. When people cross the square she makes no effort to give them space to pass through. Another 2 friends join her in line, again she does not keep a distance from them.<br>3. M was alone, approaches from the library and gets in line at the first food stand. He keeps about a meter distance from the person in front of him in line. When a group of people arrive and want to pass behind him he takes a small step forward, he keeps stepping back and forth, doesn't seem to know quite where to go as he is now closer to the person in front of him. Takes another step backwards when they have passed. When someone gets in line behind him he seems to notice this at first and feel uncomfortable about it but after a while he seems to have forgotten this and stays still and looks ahead. When he walks away he does not encounter anyone else.
	☐ 11-20	2						2	2	2					
	☒ 21-30	3				3		3		3					
	☐ 31-40														
	☐ 41-50														
	☐ 50+														
