# Supplementary material for: Applying qualitative methods to experimental designs: A tutorial for the behavioral sciences
Source: PLoS One. 2025 Jun 16;20(6):e0324936. doi: 10.1371/journal.pone.0324936 (PMC12169552; doi:10.1371/journal.pone.0324936)
Supplement: Appendix C — The translated version of the topic list as was used during the short interviews. (PDF) [file pone.0324936.s003.pdf]

Appendix C: Topic list short interviews. A Dutch version of the topic list was used during short interviews, but we offer a translated version for readability.

Introduction: Hi! Can I ask you two question for a research on the COVID-19 restrictions? It will take 5 to 10 minutes, and I can walk along with you during the interview. We do audio-record the interview, for analysis at a later time.

Thank you for agreeing to participate. Please answer the questions from the perspective of your normal day-to-day life, the questions are not focused on the situation at Utrecht Science Park.

I start the audio-recording now.

First some background questions: what study program are you a part of? In which year of the program?

- Question 1. What do you think of the COVID-19 restrictions?
- Question 2. Do you intend to adhere to the COVID-19 restrictions?

In the next few weeks some more extended, longer interviews, will be conducted in a project related to this one. Would you like to participate? This interview can be conducted at a time that suits you, face-to-face or via Teams.

I have one final question for you: How would you feel if you were being observed in this time of crisis in the context of a COVID-19 related research? Possible explanation: for instance a study that would document behavior in public spaces, in order to determine how well the COVID-19 restrictions are adhered to, and to investigate how people would react to aspects related to the restrictions.

Potential follow-up questions to ask during the interview:

- Question for clarification, for example:
  - Can you explain that?
  - What do you mean with ...?
  - How should I interpret what you just said?
  - In what way do you mean this?
- Text for elaboration:
  - With the COVID-19 restrictions we refer to the restrictions that the government takes to reduce spreading the COVID-virus. This includes the call to keep 1.5m distance, to wear face-masks in public spaces, and to limit social contact.
- Follow-up questions:
  - And how about other situations? [if the participant has a big focus on situations outside the university]
