# Supplementary material for: Applying qualitative methods to experimental designs: A tutorial for the behavioral sciences
Source: PLoS One. 2025 Jun 16;20(6):e0324936. doi: 10.1371/journal.pone.0324936 (PMC12169552; doi:10.1371/journal.pone.0324936)
Supplement: Appendix D — The translated version of the topic list as was used during the long semi-structured interviews. (PDF) [file pone.0324936.s004.pdf]

Appendix D: Topic list long interviews. We used a Dutch version during the long interviews, but offer a translated version for readability.

Topic with subtopics	Check	Questions & Follow-up questions
**Start**		Give explanation over informed consent and let them sign the form (if they haven't already).
Informed consent		Acquire relevant demographic information through informal conversation (gender, age, study program, year of study)<br><br>Give explanation regarding study: During this interview we will talk about life in times of the COVID-19 crisis. To structure the conversation we have selected some subtopics based on which we can discuss this topic. We are very much interested in the way you, as a student, experience this situation. You are encouraged to talk from your own perspective, and provide information on the context of the situation if that might be relevant.
**Studying**		How is your study currently organized?
Ability to adjust		Follow up on:<br>• Physical education,<br>• amount of contact with teachers,<br>• amount of contact with fellow students.<br>What do you think of studying in this form?<br>• Follow up on:<br>• Is the study going okay?<br>• Did it require adjustment time?
Attitude		What are your ideas regarding the governments COVID-19 restrictions?<br>• How do you feel about the amount of curbing of the restrictions?<br>• Are the restrictions in proportion to the situation?<br>• If new restrictions are announced, how you know?<br>• Are you actively trying to stay up-to-date of all restrictions and developments?<br>• Why do feel this is (not) important to do?
**Daily life**		Can you explain how your week looks like?<br>• What do you do at home?<br>• When and why do you leave home?<br>• How do you make decisions about this?
**Social life**		Can you describe how your social life looks before the COVID-19 restrictions?
Study-related contact		What does having contact with other students or teachers look like?<br>• How did you arrive at this form?<br>• How do you think others perceive this?<br>• How do other students have contact?<br>• How do you feel about this?

Atittude regarding social contact

Do you experience your social life differently now than before?

- In what way?
- How do you feel about this?
- If not, do you feel the COVID-19 restrictions affect your contact moments in a different way?

Friends

What does having contact with your friend look like currently?

- How do you feel about this?
- How do you make decisions about this?

Home situation

Can you describe your living situation?

- With whom are you living there?
- How do you experience the current situation?
- Follow up on:
  - How do you deal with the COVID-19 restrictions?
  - Is your living situation changed?
  - Have you made certain agreements?

If living in a student house, follow up on:

- Do you visit your parent's home (i.e. "homehome")
- Do you deal with this differently than before?
- Did you make any agreements with them?

Family

If living at their parent's home, follow up on:

- When, and what for, do you leave home?
- How do other family members do this?
- Did you make any agreements with them?
- Why (not)?

Study/student/sport-associations

Are you currently active in a study-, student, or sport association?

- How is that currently organized?
- How do you feel about this?

Work

Do you have a job next to your study at the moment?
If yes,

- Do you have contact with other people in your job?
- If ja, how do you feel about this?
- If no, was this different before the COVID-19 restrictions?
- How do you feel about this?

		If no,<br>• Did you have a job next to your study before COVID-19?
Mood / State of mind		Do you perceive the current "new normal" to have an effect on your state of mind?<br>• If yes, how has it changed?<br>• If no, do you think other students around your perceive an effect?<br>Do you feel that you now get happiness from other things than before the measures were announced?<br>• If yes, in what way?
Future		What have you learned from this period?<br>• Are there certain things you want to continue doing when the COVID-19 pandemic is over?<br>• What are moments that give you a lot of happiness?<br>What do you look most forward to once the COVID-19 pandemic is over?<br>• What would be the first thing you would do?
Wrapping up<br>Final question		Are there things left unsaid during this interview about this topic that should be discussed?<br>How would you feel if you were being observed in public spaces such as a train station, in a supermarket, or in other public buildings, in this time of crisis in the context of a COVID-19 related research?<br>Follow up on:<br>• Why would you feel this way?<br>• What do you think other people feel about being observed?
