# Supplementary material for: Applying qualitative methods to experimental designs: A tutorial for the behavioral sciences
Source: PLoS One. 2025 Jun 16;20(6):e0324936. doi: 10.1371/journal.pone.0324936 (PMC12169552; doi:10.1371/journal.pone.0324936)
Supplement: Attachment — Submitted filename: Response to Reviewers.docx [file pone.0324936.s005.docx]

March, 2025

Dear Dr. Velez,

Thank you for giving us the opportunity to submit a revised draft of the manuscript originally entitled ‘A qualitative evaluation of an experiment: Studying the effects of empathy-inducing probes on distancing during COVID-19 to derive methodological guidelines’, PONE-D-24-29638, for consideration in PLOS ONE. We appreciate your time and effort to provide feedback on our manuscript. The reviewers have provided valuable insights in the way readers perceive our paper, which provided us with the chance to further improve the manuscript. The corresponding changes are indicated in red text (for additions) or with strikethrough (for parts that are deleted) in the manuscript. Please see the table below for a point-by-point response to the suggestions of the reviewers. All page numbers refer to the revised manuscript file: ‘Manuscript’.

The authors of the qualitative study, the worked example in our paper, have decided to make the data openly available. We have added the relevant information in the submission, with a [link](https://osf.io/e4zfw/) to the repository (<https://osf.io/e4zfw/>).

Thank you for your consideration and we look forward to your reply.

Hidde Leplaa

| Reviewer | Comment | Response |
| --- | --- | --- |
| Rev 1 | The article submitted for my review is entitled “A qualitative evaluation of an experiment: Studying the effects of empathy-inducing probes on distancing during COVID-19 to derive methodological guidelines”. The article is classically structured and clearly argued. The theme of the article is stimulating: designing a qualitative evaluation method for a quasi-experiment. Nevertheless, I feel that certain elements of the article need to be improved or supplemented. | We thank the reviewer for their kind words regarding the theme of the paper, as well as the structure and arguments in the manuscript. Before responding to all of the comments one by one, we have a more general response as well.  From the questions and comments of reviewer 1, it is clear to us that we need to better outline the main goal of this study and paper. By doing that we trust that this also solves many of the more specific comments but of course we will also still address them one by one.  The goal of this paper is to show (quantitative) researchers in the behavioral sciences how to use qualitative methods in the context of experimental research. We use the paper of Glebbeek et al. (2024) as a worked example, which was executed in the larger context of research by De Ridder et al. (2020). In our manuscript, we summarize the steps needed to execute a qualitative analysis in an experimental study. The aim of this paper, therefore, is to serve as a methodological tutorial. It’s developed by describing and discussing the methodological steps and considerations of the empirical example (Glebbeek et al) that is used as illustration throughout the paper.  To bring this structure and the scope of our paper across we made several changes to the manuscript: 1) we changed the title, 2) we have rewritten the abstract, 3) throughout the introduction we rephrased the scope of the paper (mostly on p.2 and p.4-5). |
|  | 1. In the introduction, despite the authors' didactic efforts, the distinction between experience and quasi-experience is not clear. The authors do not give a clear definition of quasi-experience and what distinguishes it from experience. This is important, however, as the originality of the article seems to rest on the study of a quasi-experience. | On p.2, in the second paragraph, we reformulated the definitions of quasi- en true experiments to better clarify the distinction.  As outlined above, we made several changes to better communicate the scope of this article and its originality. Since this is not the presentation of a quasi-experiment we made additional changes in formulations and explanations. In most places, we replaced the term “(quasi-) experiments” (comprising both options) with *experimental designs* (also comprising both options) and are explicit about when we mean true experiment or quasi-experiment. We added an explanation on p.6, that, while our illustrative example concerns a quasi-experiment, the methodological guidelines we present in this manuscript can also be applied to other experimental designs. |
|  | 2. Still in the introduction, the authors write “there is little literature on using a qualitative approach for the evaluation of experimental designs in behavioral science.” Firstly, I don't really agree with this peremptory assertion. Numerous books or articles deal to a greater or lesser extent with the qualitative evaluation of experimental design in behavioral science (for example: [1]Kite & Whitley, 2012. Principles of research in behavioral science; [2]Orcher, 2016. Conducting research: Social and behavioral science methods; [3] Maxwell 2008. Designing a qualitative study The SAGE handbook of applied social research methods; etc.). Secondly, there are a multitude of methods for evaluating qualitative experiments ([4]Rossman & Wilson, 1985, Numbers and words: Combining quantitative and qualitative methods in a single large-scale evaluation study. Evaluation review, 9(5), 627-643; [5] Horsburgh, 2003, Evaluation of qualitative research. Journal of Clinical Nursing (Wiley-Blackwell), 12(2); [6] Hennink, et al (2020). Qualitative research methods. Sage; [7] Yadav, 2022. Criteria for good qualitative research: A comprehensive review. The Asia-Pacific Education Researcher, 31(6), 679-689. etc.).   2B. I fail to see where the contribution of the new method proposed by this article lies. It should be clarified in a very precise and documented way. In particular, it should be specified whether the proposed method concerns quasi-experiments or experiments, whether it concerns mixed or totally qualitative methods, or even experiments that are mainly quantitative but incorporate qualitative data; otherwise, it's hard to see where the article's contribution lies. At the end of the introduction, the authors state “we provide a step-by-step approach step-by-step approach for future researchers wishing to carry out a qualitative evaluation of a"quasi-)experimental design”, but it is not clear whether this is really a research gap. In this respect, in the “Methodological approach” section, the authors state “Our own study is an example of adding a qualitative element to a quantitative study”, so we'd be tempted to believe that the gap lies here, but we'd have to justify it much earlier in the article. | Thank you for providing suggestions for relevant literature. We were familiar with almost all, and checked the other references. While this work is indeed about qualitative research, it lacks the specific methodological focus on the qualitative evaluation of experimental designs in behavioral sciences.  We added several of the suggested references for further reading on qualitative methods in general (p.4). Also the references about quantifying qualitative results that were suggested by the reviewer, are now included (p.4).  2B. We trust that with a better presentation of the scope of this paper (see first response), and a better positioning with regards to type of designs (see response to remark 1), we have now clarified what the contribution of this paper is. |
|  | 3. Still in this “Methodological approach” section, the authors mention a large number of bibliographical references (references 20 to 61). However, in a research article, bibliographical references usually appear in the introduction or discussion, but not in the methodological or results section. This gives the impression that your work is more like a literature review than a concrete experiment. The authors should clearly state their position on this point. Is the article a methodological review or the testing of a qualitative experimental method? This is not very clear. | Thanks again for pointing out that the scope of the paper was really not clearly presented. We prefer the term methodological tutorial over methodological review but, indeed, the main message is not about the results of the illustrative example we used; and thus the manuscript does not follow the typical structure of a paper presenting empirical research. See also our first general response.  With regards to questions 3, 4 and 5: We do realize that we were also using confusing language to refer to the different studies that play different roles in this work. This manuscript presents a methodological tutorial. The worked example, is the qualitative study that evaluates a quasi-experiment presented in Glebbeek et al. The study by Glebbeek et al. is part of a larger study presented by De Ridder. The different studies are now explicitly and consistently referred to as, respectively, “our study” (the tutorial), the worked example/illustration/Glebbeek et al. (the empirical qualitative study) and the bigger context study/De Ridder et al. (providing the study design and experimental manipulation). |
|  | 4. In the “data collection” section, you state that you have carried out interviews and observations, but it's hard to understand why you are doing so (for what purpose) and with whom (list of interviewees). What's more, if I understand correctly, the purpose of these observations and interviews is to justify the results of the method you describe, but your results contain very little verbatim information about these observations and interviews. So I don't see how these observations and interviews validate your method. | We feel that this remark follows from the same misconception about the general scope and the role of the empirical example in this paper. In the paper by Glebbeek et all., the empirical study and its results are presented in detail. Here, we just use it as an illustration to present a (relatively unknown and not often used) method. See also response 3.  As research methodologists with expertise and experience in qualitative methods, we feel we are in the position to write this tutorial, outlining how experimental designs can be evaluated using qualitative data and analysis. The aim is not to validate a method through an example study. |
|  | 5. Still in the presentation of the method, you insert a few verbatims after figure 2 and then announce that “Observations and interviews clearly showed that social interactions are context-dependent, i.e. that behavior depends not only on intentions (based on reasoning about actions and motivations for complying with them) but also, for example, on how friends or other contacts behave, their intentions and the perceived risk of not behaving as intended (see figure 2).”. It's possible to agree with this statement, but I don't see how it validates your method of evaluating a qualitative experiment? In my opinion, to validate a method, it is necessary to provide very factual evidence, for example by comparing the responses of the experimental group and the control group, and by showing that each stage of the method improves the results. In my opinion, your article fails to do this. Table 2 compares the results between the two groups, but I fail to see how this validates your method. To validate your method, it would probably have been more appropriate to set up two groups, an experimental group studied using the new method, and a control group not studied using this method. | We assume this comment is resolved by the changes as discussed in our first general response, combined with the responses for 3 and 4. |
|  | 6. To conclude the presentation and justification of your method, you write that “Our intention was to demonstrate the iterative process of hypothesis generation and validation through repeated re-evaluation of the data. of data.” Firstly, I don't think this intention corresponds to the initial objective of your study set out in the introduction. Secondly, I discover that your method ultimately consists in determining hypotheses by re-evaluating data. Figure 1, which presents your method, and Table 3 do not mention the construction of these hypotheses, which, in my opinion, is part of a qualitative abductive research methodology not presented in this work. | We thank the reviewer for pointing out the confusing use of the word hypothesis in this part of the manuscript. To better convey our message we replaced the term ‘hypothesis’ with ‘preliminary explanation’, as we agree that ‘hypothesis’ can be misleading in this context. The iterative process of formulating ideas on potential themes and relations between themes, and checking/testing those ideas with the other available data is inherent to the processes of axial and selective coding, both included in Table 3.  By replacing the term, and defining the replacing term on p.26, we trust this point gets across more clearly now. |
|  | 7. I'm not very convinced by your discussion, as you fail to demonstrate the validity of your method. You write “Table 3 summarizes the essential elements for conducting a rigorous qualitative investigation of potential research projects.” Table 3 presents a literature review of the various stages (Research objective; Data collection   Personally, I regularly conduct qualitative studies and apply these steps from the literature, but I've never tested the validity of the overall method, and I don't know if the method is valid. After reading your work, I still don't know. You have carried out a literature review of the design stages of a qualitative study, which is interesting, but it is not possible to say, as you do, that this method is rigorous and reproducible. To write that, it would have been necessary, at the very least, to apply this method to several studies, or to analyze the results between an experimental group and a control group. I'm not saying that your method isn't correct, but you don't provide proof of it. | See our earlier responses, especially for comment 4. Testing the validity of a method is not the scope of this paper. We aim to provide a methodological tutorial on applying qualitative methods in the context of experimental designs. |
| Rev 2 | 1. Abstract: Please include the qualitative research design, specifying that the qualitative evaluation was conducted using Grounded Theory. I would also suggest clarifying this earlier in the manuscript to help guide the reader (e.g., a guide for a qualitative evaluation based on Grounded Theory). | Thank you for pointing this out. We agree and added this to the abstract (p.1) and in the introduction at an earlier stage (p.4). |
|  | 2. A. In your manuscript, you frequently refer to the 'effect of the intervention' or 'intervention effect' in the context of the qualitative approach. The term 'effect' is more commonly associated with quantitative research, although it is debated in the literature. I find your approach very interesting, particularly in the chapter 'The intervention effect,' which, among other things, addresses the quantitative representation of qualitative results and hypothesis generation. However, please clarify more clearly, what you mean by this in the qualitative context.   2B. You often discuss how qualitative approaches enrich quantitative (quasi-experimental) studies, and at times you refer to the 'intervention effect' as something that could potentially replace quantitative approaches. In this context, I would like to refer to the sentence on page 8: 'Note that intervention effects can be evaluated exclusively through qualitative data, or the qualitative data can be an addition to quantitative data within the same study.' There may be a language issue here, but I believe this statement requires further clarification. In my view, qualitative research cannot replace quantitative intervention research, but it can certainly enhance it by providing a clearer understanding of why an intervention works or doesn't work. | 2A: We used the term intervention effect to describe any change that is assumed to be the consequence of being exposed to an intervention/manipulation, irrespective of how this change is defined or measured (i.e., with quantitative outcomes/variables or in a qualitative way). However, we agree that readers may interpret the word ‘effect’ only as a quantitative measure. We therefore added such an explanation on p.3.  2B: First of all, we see how our phrasing could be confusing, and adjusted it (p. 8). Obviously, we agree with the reviewer that qualitative experimental research cannot *in general* replace quantitative experimental research. However, we do believe and argue that one could, on occasion, decide to focus solely on qualitatively defined and measured effects. |
|  | 3. In the ethics statement and data availability section (provided by the submission system), you mention that you obtained permission to describe the data and conduct of the qualitative study by Glebbeek et al. (2024) in this manuscript. You also note an overlap in authorship, which leads me to conclude that some of the authors were involved in both the Glebbeek study and this manuscript. Furthermore, in the manuscript itself, you mention that there was a quantitative study (De Ridder) and a qualitative one (Glebbeek) related to the COVID-19 intervention, with the latter being discussed in this manuscript. That was clear to me. However, the sudden use of phrases such as 'The research goal we defined for the qualitative study…,' as well as 'In our study...' and 'We did...' in the context of the qualitative study was somewhat confusing. I suggest clarifying the distinction for the reader by describing this issue more clearly in the manuscript itself. | We completely agree and have made changes throughout the manuscript to resolve this issue.  We recognize that our language was unclear when referring to the different studies that play distinct roles in this work. This manuscript presents a methodological tutorial. The worked example is the qualitative study that evaluates a quasi-experiment presented in Glebbeek et al.. The study by Glebbeek et al. is part of a larger study presented by De Ridder.  The different studies are now explicitly and consistently referred to as, respectively, “our study” (the tutorial), the worked example/illustration/Glebbeek et al. (the empirical qualitative study) and the broader context study/De Ridder et al. (providing the study design and experimental manipulation). For a more detailed explanation, we refer to the very first response to reviewer 1 in this letter. |
|  | 4. Worked example, page 6: How long, or how many weeks, was an A or B sequence in the A-B design? | We added the information about the length of the sequences/studies (p.5). |
|  | 5. The Table 3 is great because it provides an excellent overview of the topic. One suggestion I have is to introduce an additional column and separate the references into those used for your grounded theory and those that can also be applied (as further reading). Furthermore, I understand that you cannot cover all possible qualitative aspects here, but I would like to point out that qualitative content analysis is also a viable option to be applied in this context. | Thank you for this suggestion for Table 3. We fear that an extra column does not make the overview more clear. However, in the row Data collection we added a remark that the references apply to all approaches one can choose. In the row Data analysis we clarified that we provide information/references for a CGT approach.  We also added content analysis as extra option in the row Data collection. |
|  | 6. Discussion, Limitation, the following sentence: 'Even when invited for the interview near the intervention location, most people preferred the offered option of doing the interview online or at an other time. This changed their status to control group participants.' Please explain why their status was changed to that of the control group. | The control group was asked to participate in an interview during a control week, or, at a control location (not near the intervention). The intervention group was invited to the interview near the location of the intervention, at the time when the intervention was taking place. However, if people invited to participate preferred to have the interview at another time (so not shortly after being exposed to the intervention) and/or online (other time and other location) we assumed their circumstances were more similar to the control group condition than to the intervention group condition.  We have also added this explanation to the paper on p.29. |
|  | 7. Discussion: I would find it interesting if you could revisit and discuss the two research approaches using the studies of Reed 2021 and Geed (red: Reed?) 2024. You could highlight the benefits that this qualitative study brings to the quantitative results of Reed. | We regret to say that we did not find the references suggested by this reviewer. However, given the scope of the paper, we are not convinced that a comparison with two specific quantitative studies is something we should adopt in the discussion.  If the reviewer disagrees, we are of course willing to reconsider, but we would first need the full references for these papers. |
